# A Descriptive Study of Operational Data for a Novel Early Psychosis Intervention Program for Youths Aged 12–18

**DOI:** 10.1192/bjo.2025.10475

**Published:** 2025-06-20

**Authors:** Chee Hon Chin, Li Keat Oon, Charmaine Tang

**Affiliations:** Institute of Mental Health, Singapore, Singapore

## Abstract

**Aims:** The Early Intervention in Psychosis (EPIP) team in Singapore extended its remit to see 12–15-year-old patients presenting with diagnosed psychosis (not ARMS) in 2019. This program has been running for 4 years and a sizeable data set is now available on this group of patients. This is a novel service. Research and evaluation of this service will add to the understanding of how to configure services for this clinically challenging population.

**Methods:** De-identified operational data is available at the Institute of Mental Health. Data from 2019–2022 was extracted with the permission of the Data Protection Officer and de-identified through the Data Science Office. Patients aged 12–18 seen by EPIP from 2019–2021 were included in the study. This will allow 1 year’s data to be included and studied. Descriptive statistics looking at the demographics, orders, chargeable contact points with the hospital, admissions and payment information are described for this group.

**Results:** 78 patients were found from this dataset to have been included in the 12–18 EPIP program. In the 3 years 2019–2021, there were gradually increasing numbers of patients seen in this program, 21, 25 and 32 respectively. There were similar numbers of patients in the 12–15 age group as compared with those accepted into the service between 16–18. There was a higher representation of ethnic minorities and females into the program.

**Conclusion:** This is a new service looking at confirmed cases of psychosis in the younger age group 12–15. As an estimate, this figure is similar to the number of patients accepted to Early psychosis intervention programs aged 16–18. There is an over representation of females and ethnic minorities in this clinical population. There is a distinct need for services targeting this group of patients.

The effort required for analysis of operational data is high and dependent on the quality of the operation data repository. The current state of the data sets in IMH are not conducive for studying and may limit the reliability of the data presented here. Knowledge of the dataset and its clinical implications was required to be able to process the data. Further exploration of this data is planned.

